# The Orthotic Effects of Different Functional Electrical Stimulation Protocols on Walking Performance in Individuals with Incomplete Spinal Cord Injury: A Case Series

**DOI:** 10.46292/sci23-00021S

**Published:** 2023-11-17

**Authors:** Shirin Tajali, Stephanie N. Iwasa, Vivian Sin, Sharmini Atputharaj, Naaz Desai (Kapadia), Kristin E. Musselman, Milos R. Popovic, Kei Masani

**Affiliations:** 1 The KITE Research Institute, Toronto Rehabilitation Institute – University Health Network, Toronto, ON, Canada; 2 CRANIA, University Health Network, and University of Toronto, Toronto, ON, Canada; 3 Department of Physical Therapy, University of Toronto, Toronto, ON, Canada; 4 Institute of Biomedical Engineering, University of Toronto, Toronto, ON, Canada; 5 Krembil Research Institute, University Health Network, Toronto, ON, Canada

**Keywords:** functional electrical stimulation, orthotic effect, spinal cord injury, walking

## Abstract

**Background:**

Functional electrical stimulation (FES) of paralyzed muscles can facilitate walking after spinal cord injury (SCI).

**Objectives:**

To test the orthotic effects of different FES walking protocols on lower joint kinematics and walking speed.

**Methods:**

Three adults with incomplete SCI participated in this study. Their lower extremity motor scores and 10-meter walk test results were as follows: subject A: 50, 1.05 m/s, subject B: 44, 0.29 m/s, and subject C: 32, 0.27 m/s. Participants completed four conditions of over-ground walking including no FES and three bilateral FES-walking protocols as follows: multi-muscle stimulation (stimulation of quadriceps and gastrocnemius in the stance phase, and hamstring and tibialis anterior in the swing phase), drop foot (tibialis anterior stimulation), and flexor withdrawal (common peroneal nerve stimulation). The FES system obtained gait phase information from foot switches located under the individuals’ heels. Three-dimensional kinematic analysis was undertaken to measure minimum toe clearance (MTC); ankle, knee, and hip range of motion (ROM); stride length; and stride speed.

**Results:**

Compared to no-FES walking, MTC increased during drop foot (all subjects), flexor withdrawal (subjects A and B), and multi-muscle stimulation (subjects B and C) protocols. A significant decrease in ankle ROM was seen with drop foot (all subjects), flexor withdrawal (subjects A), and multi-muscle stimulation (subjects A and C) protocols. Hip ROM increased with drop foot (subjects B and C), flexor withdrawal (subject B), and multi-muscle stimulation (subject C) protocols.

**Conclusion:**

Three FES walking protocols induced positive kinematic changes as indicated by increased MTC, decreased ankle ROM, and increased hip ROM during walking in subjects with incomplete SCI.

## Introduction

Following spinal cord injury (SCI), people can live with a range of motor disabilities due to paralysis of the upper limb(s), trunk, and/or lower limb(s).^1^-^3^ Approximately 63% of individuals with SCI are unable to walk independently after injury.^1^,^2^ These impairments often affect their function (e.g., standing, walking, and grasping objects) and participation in daily life activities (e.g., housekeeping, leisure activity, work).^1^,^2^,^4^-^8^ Today, it is well established that initiation of early and intensive rehabilitation after central nervous system (CNS) injury can dramatically impact neurological and functional recovery.^9^-^11^ Among the existing modalities for rehabilitation after SCI, functional electrical stimulation (FES) is used to produce muscular contraction to specific groups of muscles to maintain/increase muscle quality, reduce gait deviations, and stimulate neural plasticity and recovery.^1^,^6^-^8^,^12^-^15^

FES can be used to generate functions such as grasping and walking in individuals with CNS injury using either surface (transcutaneous) or subcutaneous (percutaneous or implanted) electrodes.^1^,^6^-^8^ Transcutaneous FES systems are widely used in rehabilitation as they are noninvasive, safe, easy to apply, and inexpensive.^6^-^8^,^12^,^16^ However, higher-intensity pulses are required for stimulation with transcutaneous electrodes due to the electrode-skin and skin-tissue impedance as compared to the subcutaneous electrodes.^16^,^17^ Generally, transcutaneous FES systems to improve walking are divided into two categories.^1^,^6^,^8^ The first category consists of devices that target drop foot by stimulating the muscle (tibialis anterior) or the nerve (peroneal nerve) responsible for ankle dorsiflexion.^1^,^6^,^15^,^18^ Peroneal nerve stimulation is used to elicit a flexor withdrawal reflex to simultaneously enhance hip flexion, knee flexion, and ankle dorsiflexion.^1^,^15^ However, rapid habituation has been reported upon repeatedly stimulating this reflex arc.^15^ The second category of FES systems is multi-channel stimulators designed to activate multiple muscles.^1^,^16^ These stimulators have the potential to facilitate multi-muscle functions such as walking in individuals with more profound walking impairments (e.g., impaired knee and hip flexion, in addition to limited ankle function).^1^,^14^,^19^,^20^

To date, the positive effects of multi-muscle stimulation protocols on the improvement of upper and lower limb functions have been discussed in the literature.^8^,^14^,^15^,^18^ We have demonstrated the effects of a multi-muscle FES protocol on walking function in individuals with chronic motor incomplete SCI.^14^,^15^,^18^ Our findings revealed that walking function can be improved (i.e., increased walking speed, stride length, and step frequency) by targeting major lower limb muscles including quadriceps, hamstrings, dorsi-flexors, and plantar-flexors.^15^ Later, through a randomized controlled trial, we found the benefits of a multi-muscle FES walking protocol on self-reported mobility as determined by the mobility subscore of the Spinal Cord Independence Measure.^14^ However, in the aforementioned studies,^14^,^15^ two separate stimulators with open loop controllers were used to control the gait sequence of each leg, and the gait was controlled by the therapist or patient pressing the push button shortly after heel-off phase of the gait cycle.^13^-^15^

Our team has recently developed a new closed-loop, multi-channel stimulator, using input from switches to control stimulation in a synchronized fashion during walking. MyndMove^®^ offers different stimulation protocols designed to address distal and proximal muscle impairments. New stimulation pulses have been used in MyndMove stimulators that have a very fast slew rate that can maximize the engagement of Aα efferent nerve fibers and minimize activation of nerve fibers responsible for the transmission of pain (Aδ and C).^16^,^19^,^21^ This new stimulation technique has resulted in a considerable reduction in the intensity of stimulation required to achieve motor responses and the discomfort experienced during stimulation.^16^,^19^,^21^,^22^ MyndSearch is the research version of MyndMove, and using this stimulator we have developed walking stimulation protocols (multi-muscle stimulation, drop foot, and flexor withdrawal). The purpose of the present study was to test the orthotic effects of different FES walking stimulation protocols in three individuals with incomplete SCI. We hypothesized that the three FES walking protocols could increase toe clearance, knee and hip range of motion (ROM), stride length, and speed as well as decrease plantarflexion and ankle ROM in three individuals with incomplete SCI. We also hypothesized that activating proximal muscles (quadriceps and hamstrings) in addition to ankle muscles (tibialis anterior and gastrocnemius) would improve walking in individuals with incomplete SCI who have more profound muscle weakness.

## Methods and Materials

### Subjects

Three adults with incomplete SCI participated in the research program. This study was approved by the Research Ethics Board at the University Health Network. All subjects signed consent forms detailing the experimental protocol and method before participating in the study. We included individuals with an incomplete SCI, at the level of T12 and above, and with the American Spinal Cord Injury Association Impairment Scale (AIS) of C-D. Participants were required to have the ability to stand (with a gait aid if needed but no physical assistance from another person) for at least 2 minutes. Individuals were excluded if they had any muscular and/or neurological conditions impacting mobility apart from their SCI, any types of implanted electronic devices, skin lesion at a site of stimulation electrodes or motion sensors, cancer or radiation in the past 6 months, uncontrolled autonomic dysreflexia, or botulinum toxin injection into muscles targeted by the stimulation in the past 6 months.

### FES walking protocols

**Figure 1.**
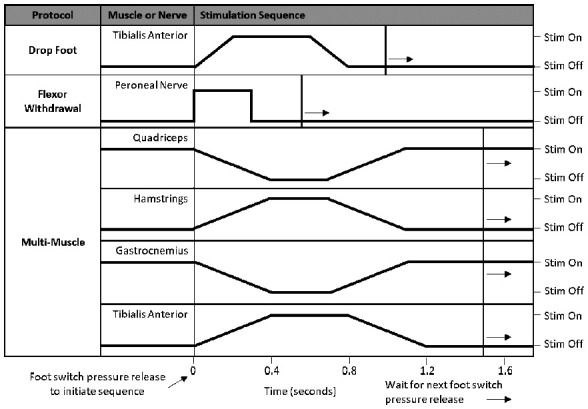
Stimulation sequence for different functional electrical stimulation (FES) walking protocols. Foot-switch pressure release initiates the stimulation sequence from the swing phase.

FES was applied during walking using the MyndSearch stimulator (MyndTec Inc., Mississauga, ON, Canada), with eight programmable stimulation channels, four analog inputs, and four digital inputs. Stimulation was applied at a constant stimulation frequency of 40 Hz and pulse duration of 400 μsec using biphasic asymmetrical pulses. The FES system obtained gait phase information from the two foot switches located under the individuals’ heel. **Figure 1** shows the stimulation sequence for different FES walking protocols used in this study. For the multi-muscle stimulation protocol, four muscle groups were stimulated on each side, including the quadriceps, hamstrings, gastrocnemius/soleus, and tibialis anterior. The stimulation sequence was delivered as follows; the quadriceps and gastrocnemius/soleus were stimulated continuously during the mid-stance and late-stance phases while the hamstrings and tibialis anterior received no stimulus. When the subject was about to initiate the swing phase (approximately 300 ms before toe-off), foot switches were released that triggered the stimulation of hamstrings and tibialis anterior. In the drop foot protocol, the heel-off caused foot switch release resulting in tibialis anterior stimulation to facilitate ankle dorsiflexion during the swing phase of gait. In flexor withdrawal protocol, the common peroneal nerve was the target of stimulation to enhance hip and knee flexion and ankle dorsiflexion during the swing phase of gait. If the foot switches were not activated due to the participant's gait, a finger switch was used by the experimenter to activate the stimulation.

### Experimental procedure

Consenting study participants first attended a screening visit where the study team members determined their eligibility for the study by assessing their baseline abilities. During the screening visit, participants were asked to complete a blood pressure reading test and a 10-meter walk test (10MWT) at their preferred speed. In addition, Lower Extremity Motor Score (LEMS) was assessed in this session. This screening session lasted for approximately 1 hour.

**Figure 2.**
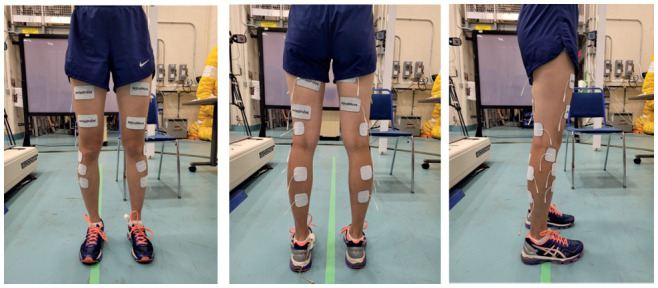
Electrode attachments for different FES walking protocols.

In the second session, subjects were asked to walk for 6 to 10 passes of a 4-meter walkway with and without FES at their preferred walking speed. **Figure 2** shows the placement of electrodes in the standing position. The surface electrodes (MyndTec Inc., Mississauga, ON, Canada) were placed bilaterally on the subject's skin over the muscles targeted with FES while the subject was in the seated position (to prevent fatigue). For the quadriceps stimulations, two self-adhesive electrodes (5 × 9 cm) were placed on the anterior and the middle of the thigh where there was maximum muscle bulk. For hamstrings stimulation, electrodes (5 × 9 cm) were placed on the posterior and the middle of the thigh. For gastrocnemius muscles, electrodes (5 × 5 or 5 × 9 cm) were placed on the proximal and distal parts of the calf. For the tibialis anterior, electrodes (5 × 5 cm) were placed on the proximal and distal anterior aspect of the lower leg, lateral to the shin of the tibia. For all muscles, the active electrode was placed proximally, and the reference electrode was placed distally. For the flexor withdrawal protocol, the active electrode (5 × 5 cm) was positioned just below the fibular head and the reference electrode was positioned in the popliteal fossa laterally. In addition, retro-reflective markers were placed bilaterally on the acromion process, anterior superior iliac spine, posterior superior iliac spine, greater trochanter, lateral condyle of femur, shoe's heel, and anterior toe regions.

After attaching the electrodes and markers and prior to the initiation of data collection, the stimulation intensity was determined for each participant and each muscle by gradually increasing the stimulation intensity (1 mA increments) until the desired motion was attained and no further increase in muscle contraction occurred. At the beginning of data collection, participants were asked to walk at a comfortable speed without FES (no-FES condition). Then, participants were randomly assigned to complete three FES walking protocols including multi-muscle, drop foot, and flexor withdrawal stimulation. Subjects completed all walking experiments on a walkway. To minimize the risk of falling, a safety harness attached to the ceiling was provided to each participant during the training session. In addition, a physiotherapist supervised the participants closely to provide assistance in case of a loss of balance. For the purpose of familiarization, participants practiced each FES walking protocol before starting data collection. Rest time, if needed, was given to participants between each condition to minimize fatigue (5-15 minutes). Time was also spent between conditions setting up the next protocols, which could help give participants a break.

### Kinematic data processing

Three-dimensional gait analysis was undertaken using 250 Hz 10 infrared cameras (Raptor-E, Motion Analysis, CA, USA). Marker data was analyzed offline using a custom-written script in MATLAB (MATLAB 2020a, MathWorks, Natick, MA, USA). Data were filtered using a fourth-order Butterworth filter with a cutoff frequency of 4 Hz. Joint angles were calculated using shoulder, hip, knee, and toe markers. To parse the data, heel strike was identified as the maximum distance between the swing foot (heel) and stance foot as reference (ankle) in the x direction. Derivatives of the distances were calculated, and zero crossings were identified. The final heel strike index was verified manually. The onset of heel off was defined as the point when heel marker z position is 3 *SD* above the mean, where the mean is based on the first 20% of the gait cycle. Stride length was defined as the absolute difference between the two heel markers across subsequent steps. Minimum toe clearance (MTC) was defined as the minimum vertical distance between the lowest point of the toe marker and the ground during the early swing phase. Ankle, knee, and hip ROM were calculated between heel off and heel strike using maximum and minimum angle values. The average value of each of these angles was calculated over the 6 to 10 trials for each participant and each condition.

### Statistical analysis

Statistical analysis was performed using SPSS version 26.0 (IBM, Inc., Chicago, IL, USA). The Kolmogrove-Smirnoff test was used to test the normality of outcome measures. A one-way repeated measure analysis of variance (ANOVA: four conditions: no-FES, and three FES walking conditions: multi-muscle, drop foot, and flexor withdrawal) was conducted to compare differences in MTC; ankle, knee, and hip ROM; stride length; and speed in each participant. A post hoc test with Bonferroni correction was performed in the case of significance. Partial eta squared (η^2^) was used to compare the effect sizes with the values ranging from 0.01-0.06, 0.06-0.25, and >0.25 indicating small, medium, and large effect sizes. A *p* value of ≤.05 was considered statistically significant.

## Results

**Table 1** shows the demographics and the clinical characteristics of our study participants. Subject A was a community walker who did not use any walking aids but reported difficulty during ambulation and gait asymmetry (especially in his left leg). Subjects B and C were limited household walkers and required mobility aids for ambulation. Subject B was using a cane on the right side for indoor mobility, which he used during the experiment. He could move independently with a manual wheelchair for outdoor mobility. Subject C required a four-wheeled walker for indoor mobility, which was used during the experiment, and a manual wheelchair for outdoor mobility. Subject C was using an ankle-foot orthosis on her left leg for indoor mobility. However, she was asked not to use her orthosis during the experiment as it could interfere with the FES effect. Subjects A and B completed three FES walking protocols including bilateral multi-muscle stimulation, drop foot, and flexor withdrawal in a session. In subject C, the flexor withdrawal protocol was not used for data collection as we were not able to elicit this reflex. We also used the finger switch to mimic the role of the foot switch during the multi-muscle stimulation protocol in subject C.

**Table 1.** Demographic and clinical characteristics of study participants

Demographic and clinical characteristics	Subject A	Subject B	Subject C
Age, years	61	47	47
Sex	M	M	F
Neurological level of injury	C4	C7	C5
AIS	D	C/D	D
LEMS	50	44	32
Hip flexors/knee extensors/ankle dorsi-flexors/ankle plantar-flexors/great toe extensors	Right:5/5/5/5/5 Left:5/5/5/5/5	Right:5/5/5/5/5 Left:4/4/4/4/3	Right:4/5/4/5/5 Left:2/4/1/1/1
10-meter walk test	1.05 m/s	0.29 m/s	0.27 m/s
Ambulation status	Community walker	Limited household walker	Limited household walker

*Note:* AIS = American Spinal Injury Association Impairement Scale; LEMS = Lower Extremity Motor Score.

**Figure 3.**
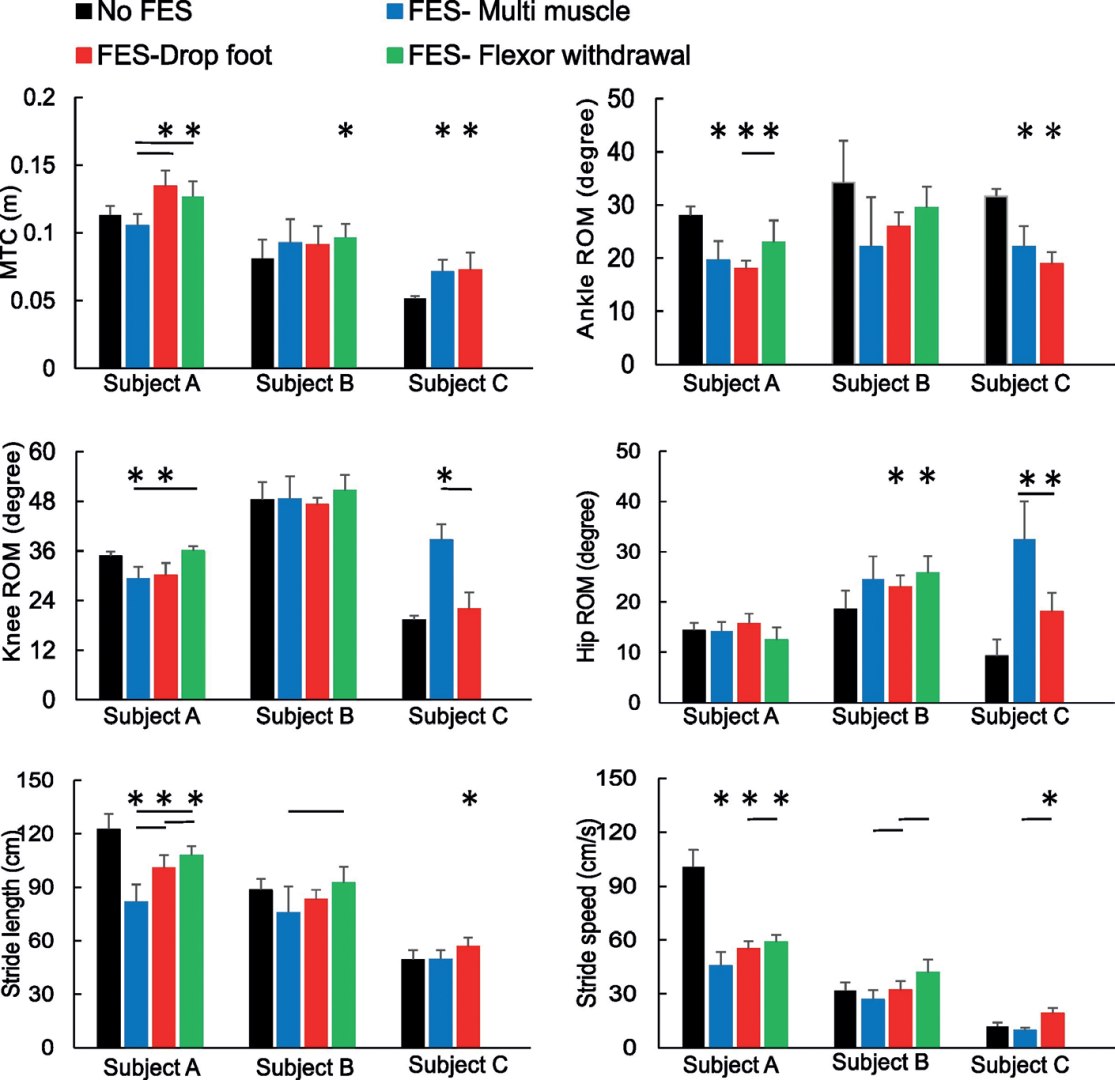
Changes in minimum toe clearance (MTC); ankle, knee, hip range of motion (ROM); stride length; and stride speed on the more affected (weaker) side during different functional electrical stimulation (FES) walking protocols in three subjects with incomplete spinal cord injury (SCI). Stars indicate a significant difference with no-FES condition. Horizontal lines indicate differences between FES-walking protocols.

**Figure 4.**
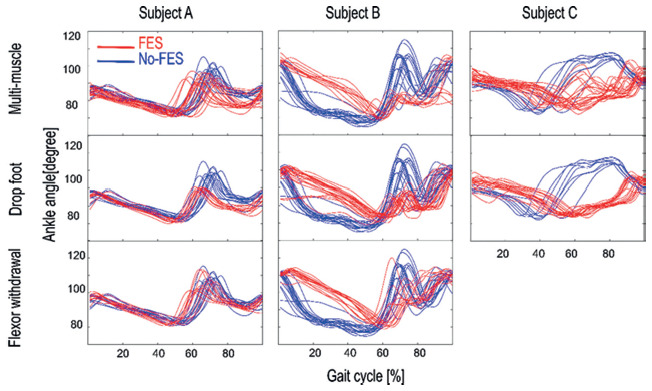
Ankle trajectories of the more affected (weaker) side during over-ground walking with three different FES-walking protocols including multi-muscle, drop foot, and flexor withdrawal in three subjects with incomplete SCI. Each trace represents one gait cycle during FES (red) and no-FES (blue) conditions.

**Figure 3** shows the orthotic effects of three FES-walking protocols on MTC; ankle, knee, and hip ROM; stride length; and speed. **Figure 4** shows ankle trajectories during the three FES and no-FES conditions on the more affected limb (left side for all three subjects). The statistical results are described in detail in the following sections for each subject.

### Subject A

As shown in **Figure 3**, MTC significantly increased with FES on the weaker side compared to no-FES (*F*_3,27_: 20.72, *p* < .001, η^2^= 0.69) in subject A. Pairwise comparison showed that MTC increased on the weaker side with the drop foot (*p* < .001) and flexor withdrawal (*p* = .03) protocols. The increase in the MTC with the drop foot (*p* < .001) and flexor withdrawal (*p* < .001) protocols was more than that observed during multi-muscle stimulation on the weaker side.

With regard to ankle ROM and in comparison to no-FES walking, different FES protocols resulted in a significant decrease in ankle ROM on both sides in subject A (right: *F*_327_: 34.71, *p* < .001, η^2^ = 0.79; left: *F*_3,27_: 20.88, *p* < ^,^.001, η^2^ = 0.69). The pairwise comparisons revealed that ankle ROM decreased with all FES protocols on the weaker side (all *p*s < .001) and with drop foot and flexor withdrawal protocols on the stronger side (all *p*s < .001). The decrease in ankle ROM was greater during the stimulation of the tibialis anterior muscle (drop foot) than peroneal nerve stimulation (flexor withdrawal) on the stronger side (*p* < .001). Analysis of the maximum ankle angle (maximum plantarflexion) in subject A showed that the maximum angle decreased on both sides with different FES-walking protocols (right: *F*_327_: 20.62, *p* < .001, η^2^= 0.69; left: *F*_1.39,12.53_: 14.13, *p* < .0^,^1, η^2^= 0.61) (**Figure 4**). Further pairwise comparisons showed that the maximum ankle angle decreased during multi-muscle stimulation (right *p* = .01, left *p* < .001) and drop foot protocols (right *p* = .01, left *p* < .001) compared with no-FES. It decreased more with the drop foot protocol compared with the flexor withdrawal protocol (*p* < .001).

In subject A, knee ROM significantly decreased on both sides (right: *F*_327_: 10.80, *p* < .001, η^2^ = 0.54; left: *F*_1.69,15.24_: 9.75, *p* <^,^ .001, η^2^ = 0.52) with FES indicating smaller knee ROM when compared with the no-FES condition (all *p*s = .01). Pairwise comparisons showed that knee ROM decreased on the right side during multi-muscle stimulation (*p* < .001) and on the left side during both multi-muscle and drop foot stimulations (all *p* < .001). Multi-muscle stimulation affected knee ROM more than flexor withdrawal stimulation on the weaker (*p* = .01) and stronger (*p* = .02) sides. Regarding hip ROM, FES did not affect hip ROM in subject A.

Subject A exhibited shorter stride length (right: *F*_1.87,18.84_: 96.47, *p* < .001, η^2^ = 0.91; left: *F*_3,33_: 49.36, *p* < .001, η^2^ = 0.84) and lower stride speed (right: *F*_1.63,14.68_: 39.96, *p* < .001, η^2^ = 0.81; left: *F*_3,33_: 133.37, *p* < .001, η^2^ = 0.93) with all FES-walking protocols. The shorter stride length and slower stride speed were found with the multi-muscle stimulation protocol in comparison with drop foot and flexor withdrawal stimulations (all *p*s < .01)

### Subject B

In subject B, MTC significantly increased on both the weaker (left) and also stronger (right) sides with FES compared to no-FES walking (right: *F*_330_: 26.19, *p* < .001, η^2^ = 0.72; left: *F*_3,30_: 2.82, *p* = .0^,^4, η^2^ = 0.22). The further pairwise comparison revealed that in comparison to no-FES, MTC increased with all three FES protocols on the right side (multi-muscle *p* < .001, drop foot *p* = .03, flexor withdrawal *p* < .001) and with flexor withdrawal (*p* = .04) protocol on the left side. The increase in MTC was greater with the flexor withdrawal compared to the drop foot (*p* < .001) and multi-muscle (*p* = .01) protocols on the right side.

With regard to ankle ROM, it decreased on the stronger side (*F*_3,33_: 5.88, *p* < .001, η^2^ = 0.34) during drop foot stimulation (*p* < .001) compared to no-FES in subject B. The decrease in ankle ROM was greater during stimulation of the tibialis anterior muscle (drop foot) than peroneal nerve stimulation (flexor withdrawal) on the stronger side (*p* < .001). Maximum ankle angle decreased more in the multi-muscle stimulation protocol compared to flexor withdrawal (*p* = .03) on the weaker side (**Figure 4**). In subject B, FES did not have any significant effects on knee ROM in comparison to no-FES. However, knee ROM was greater with flexor withdrawal stimulation compared to drop foot stimulation (*p* = .04). Regarding hip ROM, it increased on the weaker side (*F*_3,33_: 8.76, *p* < .001, η^2^ = 0.44) with the drop foot and flexor withdrawal protocols.

In subject B, there was no significant difference in stride length between FES and no-FES conditions. With regard to stride speed, the only significant difference was seen when multi-muscle stimulation was compared with no-FES walking in which stride speed decreased on the right side with FES (*F*_3,30_: 21.39, *p* = .01, η^2^ = 0.68). Pairwise comparisons in subject B showed differences between FES protocols in which stride speed was slower with the multi-muscle and drop foot stimulations than with the flexor withdrawal protocol.

### Subject C

In subject C, the changes observed in the MTC with FES were significant on the weaker side (*F*_2,14_: 12.03, *p* < .001, η^2^ = 0.63). Compared to no-FES walking, both multi-muscle and drop foot protocols increased MTC on the left side (*p* < .001). Ankle ROM decreased on the weaker side (*F*_2,14_: 45.12, *p* < .001, η^2^ = 0.86) during both the multi-muscle and drop foot protocols compared with the no-FES condition (all *ps* < .001). The maximum ankle angle also decreased on the weaker side (*F*_2,14_: 29.39, *p* < .001, η^2^ = 0.80) during the multi-muscle and drop foot stimulations (all *ps* = .01). In subject C, knee ROM increased on both sides with multi-muscle stimulation and drop foot stimulations (right: *F*_2,14_: 7.91, *p* < .001, η^2^= 0.52; left: *F*_2,14_: 88.66, *p* < .001, η^2^= 0.91). Pairwise comparisons showed greater knee ROM during multi-muscle stimulation on both sides (*p* < .001) compared with the no-FES condition compared with no-FES condition and drop foot condition on the left side (p < .001). Furthermore, hip ROM increased on the weaker side (*F*_2,14_: 39.32, *p* < .001, η^2^= 0.84) for both the multi-muscle and drop foot protocols (all *ps* < .001). The increase was greater with multi-muscle stimulation than with drop foot stimulation (*p* < .001).

In subject C, the application of FES resulted in increased stride length on both sides (right: *F*_214_: 8.91, *p* < .001, η^2^= 0.56; left: *F*_2,14_: 6.11, *p* < .05, η^2^, = 0.46). It increased with multi-muscle (*p* < .001) and drop foot (*p* = .04) stimulations for the right side, and only with drop foot stimulation (*p* = .04) for the left side. Stride speed also increased on both sides (right: *F*_2,14_: 21.96, *p* < .001, η^2^= 0.75; left: *F*_2,14_: 35.47, *p* = .00, η^2^ = 0.83) with the drop foot protocol in comparison to the no-FES condition (all *ps* < .001). Stride speed was greater with drop foot stimulation than with multi-muscle stimulation.

## Discussion

This is the first study that has investigated the orthotic effects of different FES stimulation protocols using input from switches to control FES in a synchronized fashion during walking. So far, the majority of FES studies in the field of SCI have applied FES to ankle dorsi-flexors or the common peroneal nerve to improve walking function, while only a few studies have tested the therapeutic effects of multi-muscle stimulation protocols.^1^,^8^,^14^,^15^,^23^,^24^ Furthermore, no studies have yet compared the orthotic effects of different FES walking protocols on gait kinematics in individuals with incomplete SCI. Our findings showed that the FES-walking protocols used in this study had a strong effect (η^2^ > 0.25) on improving MTC during walking in three individuals with incomplete SCI. Hip ROM mainly increased in subjects B and C who were slow walkers. The effects of different FES walking on knee ROM, stride length, and speed were not consistent, which may be due to varying degrees of muscle impairments, and differences in the baseline walking speed in our study participants.

Foot clearance is an important gait parameter that has been related to the increased risk of falling in individuals with central nervous system disorders including stroke, multiple sclerosis, and SCI.^25^-^27^ In normal gait, activation of ankle dorsi-flexors and hip and knee flexors help to clear the toe off the ground in the early swing phase.^28^,^29^ Our findings showed that the drop foot protocol that stimulates the tibialis anterior muscle was effective in improving foot clearance as measured by MTC during the swing phase of gait, suggesting that the stretch reflex of plantar-flexors may be reduced by stimulating the dorsi-flexors.^30^ Flexor withdrawal protocol also improved MTC in the two subjects except subject C who was not able to perform this condition. A previous study in persons with chronic incomplete SCI also found that a FES protocol that stimulates the common peroneal nerve helped to improve foot clearance during the swing phase in 19 individuals with incomplete SCI.^29^ Multi-muscle stimulation improved MTC in subjects B and C indicating that stimulation of proximal muscles in addition to distal muscles can affect gait function in subjects with more profound muscle weakness and impaired ambulatory function.

To the best of our knowledge, this study is the first study to look at the immediate benefits (orthotic effects) of different FES walking protocols on the lower joint kinematics in individuals with incomplete SCI. Specifically, we looked at distal (ankle) as well as proximal (hip and knee) joints kinematics during walking. Our findings showed that changes in ankle joint kinematics happened with FES as shown by reduced ankle ROM and decreased ankle plantarflexion during the swing phase of gait. In subjects A, and B, the decrease in ankle ROM was more during stimulation of the tibialis anterior muscle (drop foot) than peroneal nerve (flexor withdrawal). However, changes in hip ROM occurred only in the low-functioning SCI individuals (subjects B and C). Specifically, in subject C, multi-muscle stimulation had a strong effect on increasing hip and knee ROM. Further inspection of her clinical tests showed that this participant had no active movement against gravity (i.e., grade <3/5) in the hip flexors, ankle dorsi-flexors, plantar-flexors, and great toe extensor of her more affected side. Therefore, the multi-muscle stimulation FES protocol helped in substituting for the weakness in multiple muscle groups.

The orthotic effect of FES on stride length and speed was not consistent across our study participants. While stride length and speed decreased in subject A who had the highest walking speed (10MTW: 1.05 m/s) and ambulation level, stride length and speed increased in subject C who had the lowest baseline walking speed (10MWT: 0.27 m/s) and the most profound muscle weakness as shown by LEMS (32/50). Studies have shown that in individuals with incomplete SCI, the slower the speed of walking, the greater the effect of FES; this indicates that there is a negative correlation between gait speed and FES.^31^,^32^

In conclusion, this case series demonstrates that three FES-walking protocols used in this study were effective in immediately improving foot clearance during the swing phase of gait in 3 individuals with incomplete SCI. In subjects A and B, the decrease in ankle ROM was more prominent during stimulation of the tibialis anterior muscle (drop foot) than the peroneal nerve (flexor withdrawal). Subjects B and C appeared to benefit from stimulation of proximal muscles in addition to distal muscles during walking as shown by improved MTC and hip ROM during multi-muscle stimulation.

We acknowledge several limitations in our study. Firstly, our study participants represented a range of functional abilities ranging from participants who were limited household walkers and used an assistive device to walk (subjects B and C) to a participant who was a community ambulator without any assistive device (subject A). Secondly, lower limb kinematics were measured as the main outcome in this study, which may provide little information about the efficiency and stability of walking patterns. Future studies should subgroup participants with different functional abilities and measure different physiological and biomechanical outcomes (oxygen consumption, energy expenditure, and gait stability) to better quantify changes induced by FES across individuals with a wider range of gait abilities.
